# The role of D4 receptor gene exon III polymorphisms in shaping human altruism and prosocial behavior

**DOI:** 10.3389/fnhum.2013.00195

**Published:** 2013-05-14

**Authors:** Yushi Jiang, Soo H. Chew, Richard P. Ebstein

**Affiliations:** ^1^Department of Economics, National University of SingaporeSingapore; ^2^Department of Psychology, National University of SingaporeSingapore

**Keywords:** *DRD4*, polymorphism, prosociality, altruism, gene × environment interaction, G × E

## Abstract

Human beings are an extraordinarily altruistic species often willing to help strangers at a considerable cost (sometimes life itself) to themselves. But as Darwin noted “… he who was ready to sacrifice his life, as many a savage has been, rather than betray his comrades, would often leave no offspring to inherit his noble nature.” Hence, this is the paradox of altruism. Twin studies have shown that altruism and other prosocial behavior show considerable heritability and more recently a number of candidate genes have been identified with this phenotype. Among these first provisional findings are genes encoding elements of dopaminergic transmission. In this article we will review the evidence for the involvement of one of these, the dopamine D4 receptor (*DRD4*) gene, in shaping human prosocial behavior and consider the methodologies employed in measuring this trait, specific molecular genetic findings and finally, evidence from several Gene × Environment (G × E) studies that imply differential susceptibility of this gene to environmental influences.

## Introduction

Human beings engage in prosocial behavior, sometimes at a considerable personal cost. Charitable giving, volunteer work and even risking life and limb to save others are not uncommon. Such prosocial behavior cannot be easily explained by natural selection viz., the “selfish gene.” Not surprisingly then, the paradox of prosociality and altruism have been the subject of speculation, inquiry and even wonder from Adam Smith and Charles Darwin to the present day. Not only are the origins, motivations and mechanisms of such behavior intriguing, but also the causes underlying the remarkable individual differences in prosociality/altruism are the focus of an increasing number of studies.

Evolutionary theories have suggested various mechanisms toward understanding the origins of prosocial behavior and altruism. The *Kin selection* theory (Haldane, 1932; Hamilton, 1964a,b; Smith, 1964), for example, proposes that altruism is maintained because it increases the odds of individual gene transmission to related generations. Although this theory might help to understand altruism toward kin, it does not explain the widely observed altruistic behavior that human beings exhibit toward perfect strangers. Other hypotheses that could account for such phenomena include reciprocity and reputation building (Fehr and Fischbacher, 2003), altruistic punishment (Fehr and Gachter, 2002), and group selection (Eldakar and Wilson, 2011), among others. While these studies attempt to uncover the origins of prosocial behavior, behavioral genetics provides insights on individual differences partially hard-wired by our genomes, while contingent on the varied environmental influences organisms encounter across the life span.

Twin studies demonstrate the considerable heritability of prosocial behavior. An early study by Matthews et al. (1981) estimated the heritability of “empathic responsiveness” from a sample of adult male twins and found an estimated twin correlation at 0.42–0.72. Rushton et al. (1986) showed that ~50% of variance in altruism can be explained by genes. Although twin studies give us the sense of the genetic landscape of altruism, only molecular genetic approaches can inform regarding specific gene contributions to such behavior.

Dopamine (DA) related genes are plausible candidates for partially encoding prosociality/altruism given the functional involvement of DA transmission in approach behavior and reinforcement learning (Schultz, 2007). Among these genes, the dopamine D4 receptor (*DRD4*) gene has been examined in particular for its association with prosocial behavior, albeit with mixed results. For example, a significant association between *DRD4* and altruism has been found by Bachner-Melman et al. (2005) using the Selflessness questionnaire, and later replicated by Anacker et al. (2013) using the better known NEO-PI-R (altruism subfacet). However, other studies failed to observe a main effect of *DRD4* on prosociality whereas a Gene × Environment (G × E) interaction was demonstrated (Bakermans-Kranenburg and van Ijzendoorn, 2011; Knafo et al., 2011). This review aims to summarize the relationship between *DRD4* and prosocial behavior paying specific attention to differences in methodology and behavioral outcomes. In particular, we address the role of environments that modulate the action of *DRD4* in mediating prosocial and altruistic behavior, and discuss how these G × E interactions are crucial to understanding the behavioral impact of this gene. Importantly, we discuss various evolutionary interpretations toward a deeper understanding how this gene came into play in human altruism.

## *DRD4* exoniii VNTR

**Figure 1:**
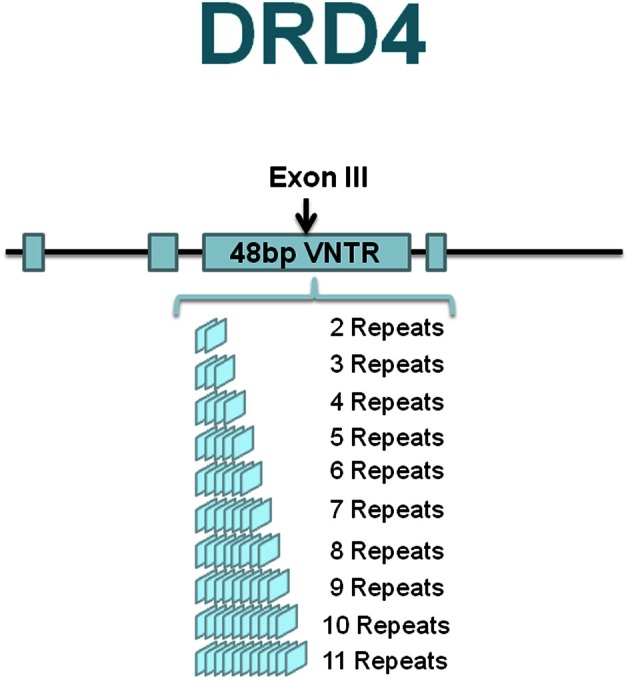
**DRD4 exonIII polymorphisms**.

The *DRD4* gene is characterized by an unusual 48-bp variable number tandem repeats (VNTR) polymorphism in the exon III coding region that codes for 16 amino acids (Lichter et al., 1993; Rondou et al., 2010). Two to eleven repeats (R) of the VNTR are observed in humans with the 4-repeat (4R) allele being the most common polymorphism (Figure 1), followed by the 7R in Caucasian populations (Van Tol et al., 1992) and 2R in East Asians (Chang et al., 1996). The 2R has been speculated as a “displacement” for the 7R in Asian populations and in this group it appears to function as the “risk” allele (Leung et al., 2005; Reist et al., 2007). Intriguingly, whereas the origins of 2R–6R alleles can be explained by simple one-step recombination/mutation events, the origin of 7R is less straightforward. Evidence suggests that this allele originated as a rare mutational event that nevertheless increased to high frequency in human populations by positive selection (Wang et al., 2004). However, more recent analysis using the massive SNP database maintains that there is little evidence for positive selection at this locus (Hattori et al., 2009; Naka et al., 2011).

Functional significance of these repeats has been suggested in many studies (Van Tol et al., 1992; Asghari et al., 1994, 1995; Schoots and Van Tol, 2003; Van Craenenbroeck et al., 2005, 2011). For example, the 7R has been linked to suppressed *DRD4* expression *in vitro* (Schoots and Van Tol, 2003). Moreover, Van Craenenbroeck et al. (2005) showed that there was a difference in the capacity of the *DRD4.2*, *DRD4.4*, and *DRD4.7* variants to be up-regulated through the pharmacological chaperone effect. In a later study, Van Craenenbroeck et al. (2011) further suggested that the polymorphic repeat variants have different relative affinities to form homo- and heterodimers. Finally, evidence also suggests that the *DRD4*.*7* allele is associated with higher reward-related ventral striatum reactivity (Forbes et al., 2007). These results imply that the repeat lengths of the *DRD4* exon III VNTR are functionally meaningful, albeit they may not be linearly related. Therefore, it is plausible that this polymorphism could reflect complex behavioral phenotypes.

Indeed, a number of studies have reported associations between the 7R (or aggregated long alleles) and increased risk for various disorders including ADHD (Faraone et al., 2001; Maher et al., 2002), Tourette syndrome (Grice et al., 1996), obsessive compulsive disorder (Camarena et al., 2007; Walitza et al., 2008), pathological gambling (Perez de Castro et al., 1997; Eisenegger et al., 2010), substance abuse (Mcgeary, 2009), bulimia nervosa (Kaplan et al., 2008), conduct disorders (Kirley et al., 2004), autism, and schizophrenia (Emanuele et al., 2010; Lung et al., 2011). Moreover, evidence also supports the associations between these *DRD4* risk alleles, especially the 7R, and certain personality traits, including increased novelty seeking (Ebstein et al., 1996), impulsivity (Eisenberg et al., 2007), as well as propensity toward financial risks (Dreber et al., 2009, 2011; Kuhnen and Chiao, 2009).

However, these associations are not always easily replicated, suggesting that the *DRD4* gene may be better conceptualized as a plasticity gene whose effect is contingent on particular environments (Bakermans-Kranenburg and van Ijzendoorn, 2006, 2007, 2011). In this view, the so-called risk alleles are not strictly linked to a definite direction of effects; rather, depend on specific environments these plasticity alleles may show either positive or negative effects. For example, individuals carrying such differential susceptibility alleles may be more prosocial when influenced by one environment, while less prosocial in another environment. In contrast, individuals without differential susceptibility alleles are altogether likely to be less sensitive to environmental influences (Sasaki et al., 2013). These ideas gain support from a recent meta-analysis by Bakermans-Kranenburg and van Ijzendoorn (2011). This study examined the cumulative evidence for association between *DRD4* exon III VNTR and rearing environments and developmental outcomes. The results demonstrated that the seemingly “vulnerable” individuals were actually more susceptible to environments, “for better *and* for worse.” The differential susceptibility of the *DRD4* exon III VNTR has been studied for various outcomes including externalizing behavior (Bakermans-Kranenburg and van Ijzendoorn, 2006; Bakermans-Kranenburg et al., 2008), attachment disorganization (Gervai et al., 2007), ADHD (Martel et al., 2011), prosocial behavior (Bakermans-Kranenburg and van Ijzendoorn, 2011; Knafo et al., 2011); unsolved loss or trauma (Bakermans-Kranenburg et al., 2011), and most recently, delay discounting (Sweitzer et al., forthcoming).

## *DRD4* exoniii VNTR and prosocial behavior

To review existing literatures on the association between *DRD4* and altruism/prosocial behavior, we systematically searched the online database of PedMed, with key words *DRD*4+Prosoical behavior; *DRD*4+Prosociality; *DRD*4+Altruism in all fields. The search resulted in a list of seven studies, all conducted within the past decade. These studies are described in Table 1.

**Table 1:** **Study characteristics (in chronological order)**.

**Study**	**Year**	Age^*^	**Ethnicity**	**Grouping**	**#Ss**	**Phenotype**	**Measure**	**G × E**
Bachner-Melman et al.	2005	n.a.	n.a.	4R vs. 7R	1006	Selflessness^1^; TPQ-Reward^2^	Self-reported questionnaire	N
Dilalla et al.	2009	3–5 y	97% Caucasian; 3% Latino	L(at least 1 ≥ 6) vs. S (both <6)	62 (28 M)	Agression; Sharing; Prosociality; Externalizing/internalizing problem behaviors	Behavior in parent-kid/peer interaction; parental questionnaires	Y
Zhong et al.	2010	M:22.5 y; SD:2.4 y	Han Chinese	2R vs. 4/4R	208 (95M)	Fairness	Ultimatum game	Y
Bakermans-Kranenburg and van Ijzendoorn	2011	M:7.4 y; SD: 0.3 y	Born in the NL	7R(+) vs. 7R(−) (both <7)	91 (43 M)	Altruism	Donating behavior	Y
Sasaki et al.	2013	M:19.3 y; SD:2.9 y	Caucasian; Asian American	(2R + 7R) vs. otherwise	178 (106F, 68 M, 4?)	Prosocial behavior	Willingness to volunteer for prosocial causes supporting the environment	Y
Knafo et al.	2011	M:43.8 m; SD:3.3 m	Israeli	7R(+) vs. 7R(−)	211	Prosocial behavior	Compliant/self-initiated/mother rated prosocial behavior: helping/emotional support/sharing	Y
Anacker et al.	2013	M:23.1 y; SD:4.5 y	Middle-European decent	7R(+) vs. 7R(−); 4/4R vs. 4/7R	786 (246M)	NEO-Altruism^3^	Self-reported questionnaire	N

Note:

* Measured in year (y) or month (m).

1 Selfishness scale.

2 TPQ-Reward: reward scale measured by TPQ.

3 NEO-Altruism: altruism subscale measured by NEO-PI-R.

The first study was conducted by ourselves (Bachner-Melman et al., 2005), and we examined the *DRD4* exon III 4R and 7R alleles for association with altruism, as measured by the Selflessness Scale (Bachar et al., 2002) and TPQ Reward dimension (Cloninger, 1987). The Selflessness Scale “*measures the propensity to ignore ones own needs and serve the needs of others*,” thus altruism (Bachner-Melman et al., 2005), whereas the Reward dimension of the TPQ taps into altruism through elements such as empathy. Significant associations have been found between the *DRD4* exon III (D4.4) and higher Selflessness scores, as well as between the 4/4 genotype and higher TPQ Reward scores. That study has recently been replicated by Anacker et al. (2013) among a European sample using the Altruism subscale of Revised NEO Personality Inventory (NEO-PI-R) (Strobel et al., 2011). Consistent with the Bachner-Melman et al. (2005) finding, their results suggested higher altruism scores in the absence of the *DRD4* 7R allele.

A robust alternative to self-report questionnaires is the experimental assessment of human altruism. For example, Bakermans-Kranenburg and van Ijzendoorn (2011) measured children's altruism using experimentally observed donating behavior. The authors hypothesized a G × E interaction between the gene and childhood attachment with parents. Indeed, the results supported the moderating role of *DRD4* exon III repeats in the association between attachment and donating behavior. Secure attachment was significantly related to more donations, but only among children with 7R allele. Interestingly, in the same year, a study by Knafo et al. (2011) used a similar paradigm to examine the interaction between *DRD4* and parenting on children's prosocial behavior. Very similar results to the Dutch study were obtained in a sample of Israeli children. Prosocial behavior in these children was examined using three measures: Compliant (in response to social requests), Self-initiated (enacted voluntarily), and Mother-rated. Parenting measures included maternal positivity, negativity, and unexplained punishment. Although no main effect of *DRD4* was observed, the G × E interaction term was significant. Positive parenting related meaningfully to mother-rated prosocial behavior, and unexplained punishment related positively to self-initiated prosocial behavior, but only among children carrying the 7R allele. To summarize, these two studies independently carried out in distinct ethnic groups strengthen the notion that *DRD4* is a plasticity gene which is sensitive to diverse parenting styles. Notably, the impact of the polymorphism on behavior is constrained by the environment.

The study by Dilalla et al. (2009) was designed to examine the combined effects of the *DRD4* gene, environmental influences due to parents and peers and their interaction. By classifying the children into *DRD4*-L (at least one allele ≥6R) and *DRD4*-S (both alleles <6R) groups, they found that *DRD4*-L children are less prosocial in sharing with each other. Moreover, their parents were less sensitive during parent-twin interaction. Additionally, there were significant G × E interactions between *DRD4* and peer behavior/parental sensitivity: children with the high-risk alleles (*DRD4*-L) are more aggressive than the low risk allele (*DRD4*-S) carriers, but only in the low-aggression environment (when peer's behaviors are not aggressive); they are also more likely to be reported as having more externalizing problems than the low risk peers, but only when they have insensitive parents.

An intriguing environmental influence of religious priming and *DRD4* genotype on prosocial behavior was recently reported (Sasaki et al., 2013). In a sample characterized by mixed ethnicity (Caucasian and East Asian), the authors grouped *DRD4*-2R and 7R alleles together as so-called risk alleles, and measured participants' willingness to volunteer (i.e., donating time) as proxy for prosociality. Again, no main effect of *DRD4* was observed, but the interaction between gene and religious priming was significant. Consistent with the concept of differential susceptibility genes, participants with “risk” alleles (2R/7R) were more prosocial than others when primed with religion, whereas they were less prosocial than people without risk alleles in the neutral priming setting.

Finally, the *DRD4* exon III VNTR has also been linked to another aspect of prosociality: the reciprocal fairness preference as measured by an incentivized economic paradigm, the Ultimatum Game (Zhong et al., 2010). In this game two players decide on how to divide an initial endowment, with the proposer states a proposal on how much to give to the responder, and the responder states a minimum acceptable amount. If the proposal is accepted (i.e., the proposer states a higher amount than the responder's minimum acceptable amount), the amount is divided accordingly; otherwise, both would receive nothing. With this Ultimatum Game, reciprocal fairness was inferred from the responders' minimum acceptable amount, with higher amount indicating more concern for fairness. Among a sample of Han Chinese subjects, due to extremely low frequency of 7R alleles, the authors following Kang et al. (2008) considered 2R as the risk allele and combined the 2/2 genotype with 2/4 genotype for comparison with the 4/4 group. A significant main effect of *DRD4* exon III VNTR on responders' behavior was observed; subjects with the 2/2 or 2/4 genotype stated lower minimum acceptable amounts than the 4/4 genotype carriers. Moreover, a three-way interaction effect was observed between gene, gender, and season of birth (SoB): non-winter born male and winter-born female subjects with the 4/4 genotype tend to have a higher minimum acceptable amount than subjects with 2/2 and 2/4 genotype. Although SoB is less clearly interpreted than some other environmental factors such as parenting, these results nevertheless support the argument that the effect of *DRD4* is largely dependent on moderating environments.

In summary, there is modest evidence that the *DRD4* exon III VNTR 7R allele is associated with diminished altruism, especially when assessed with self-report questionnaires. However, the evidence for a role of *DRD4* in altruism is stronger when the genetic effects are examined together with environmental influences. The risk alleles including 2R and the long alleles (≥6R) are shown to be differential susceptibility alleles, which contribute differentially to observed prosocial behavior contingent on environmental characteristics.

## Discussion

Based on recent evidences, our brief overview of the involvement of *DRD4* exon III VNTR in shaping human altruism/prosocial behavior underscores the notion of differential susceptibility for this polymorphism (Bakermans-Kranenburg et al., 2008; van IJzendoorn et al., 2008; Belsky et al., 2009; Bakermans-Kranenburg and van Ijzendoorn, 2011; Belsky and Beaver, 2011; Knafo et al., 2011). Whereas a main effect of the gene on prosocial behavior is not consistently observed, nevertheless when the environment is factored into the association a clearer picture appears to emerge. The risk alleles which are thought to be linked with lower prosociality can actually be more prosocial when the environment is supportive.

An evolutionary model for differential susceptibility has been suggested by Belsky (1997), in which he proposed that differential susceptibility is maintained for maximizing reproductive fitness of species in a continually changing and fundamentally uncertain environment. The variation in susceptibility to environmental influences ensures that the changes in environments would lead to diversified reactions among offspring, and thereby increase the probability of transmission of one's gene from generation to generation in an unpredictable world. We conjecture that the early migration out-of-Africa by our species unfolded as a series of unpredictable events, and this creates a favorable environment for selection of plasticity genes such as *DRD4*. Such an evolutionary argument brings us a deeper understanding of the association between *DRD4* and prosocial behavior. As hypothesized by Chen et al. (1999), and later supported by Matthews and Butler (2011), the 2R and 7R alleles of *DRD4* exon III VNTR are associated with population histories of migration. It appears that the serial migration that characterized the human out-of-Africa trek, *selects* for subjects carrying 2R and 7R alleles. Early human society in the Upper and Middle Paleolithic was characterized by small bands of hunter-gatherers, and prosocial behavior and cooperation among con-specifics would likely increase the overall fitness of such groups; this characteristic leads us to speculate that, under strict social norm/rules to promote egalitarian and prosociality within band, altruistic traits encoded in part by the *DRD4* 2R and 7R may have contributed to the remarkably successful out-of-Africa global trek beginning ~50 k ago. Hence, we hypothesize that, along with risk taking behavior, altruistic traits that are associated with the 2R and 7R exon III repeats under supportive environment partially explains the selection for these two genetic variants in the serial migration out-of-Africa that led to *Homo sapiens'* successful population of the entire planet.

The evidence that *DRD4* polymorphisms differentially contribute to prosocial behavior, can also shed light on the biological roots of human morality. Researchers have long debated regarding the mechanisms and motives underlying prosociality/altruism. Some argue that people behave in a prosocial manner because of the so-called warm glow (Andreoni, 1990), i.e., people feel good by doing good. Others suggest that it is social pressure (Dellavigna et al., 2012) that drives people to engage in prosocial behavior, due to the cost borne by disregarding peer-established norms of behavior. As argued by Sasaki et al. (2013), these two seemingly disparate conjectures may be harmonized by the differential susceptibility hypothesis, based on the role of dopamine in reward-related process (Nemirovsky et al., 2009). Warm-glow individuals, characterized by the DRD4 4/4 repeats, are “born” prosocial irrespective of the environment due to the high dopaminergic tone driven by their genotype. In contrast, carriers of the 7R risk alleles have lower baseline dopamine tone and hence are only prosocial in the presence of high environmental stimulation such as positive parenting (Wang et al., 2004). These conjectures have salient implications for parenting, moral education, policy-making and even jurisprudence. Individuals with the susceptibility alleles are theorized to be more responsive to moral education and policy interventions; to promote prosociality among this group, positive environments and rewards may be more effective than harsh environments and punishments. Conversely, for individuals without the susceptibility alleles, and thus less responsive to environmental changes, a more disciplined environment might be required to prevent deviations from societal norms of prosocial behavior.

Finally, caution needs to be exercised in interpreting existing G × E studies of *DRD4* and prosocial behaviors, since all studies to date are based on cross-sectional designs and lacking an important dynamic perspective. We do not know for example, how G × E interactions play out across the lifespan from early development to adulthood. As suggested by Bakermans-Kranenburg and van Ijzendoorn (2011), only a longitudinal design can trace the temporal interplay between the gene and the ever-changing environments that characterize our maturation and aging.

### Conflict of interest statement

The authors declare that the research was conducted in the absence of any commercial or financial relationships that could be construed as a potential conflict of interest.
